# Personality, organizational stress, and attitudes toward work as prospective predictors of professional burnout in hospital nurses

**DOI:** 10.3325/cmj.2011.52.538

**Published:** 2011-08

**Authors:** Jasna Hudek-Knežević, Barbara Kalebić Maglica, Nada Krapić

**Affiliations:** Department of Psychology, Faculty of Humanities and Social Sciences, University of Rijeka, Rijeka, Croatia

## Abstract

**Aim:**

To examine to what extent personality traits (extraversion, agreeableness, conscientiousness, neuroticism, and openness), organizational stress, and attitudes toward work and interactions between personality and either organizational stress or attitudes toward work prospectively predict 3 components of burnout.

**Methods:**

The study was carried out on 118 hospital nurses. Data were analyzed by a set of hierarchical regression analyses, in which personality traits, measures of organizational stress, and attitudes toward work, as well as interactions between personality and either organizational stress or attitudes toward work were included as predictors, while 3 indices of burnout were measured 4 years later as criteria variables.

**Results:**

Personality traits proved to be significant but weak prospective predictors of burnout and as a group predicted only reduced professional efficacy (R^2^ = 0.10), with agreeableness being a single negative predictor. Organizational stress was positive, affective-normative commitment negative predictor, while continuance commitment was not related to any dimension of burnout. We found interactions between neuroticism as well as conscientiousness and organizational stress, measured as role conflict and work overload, on reduced professional efficacy (β_NRCWO_ = -0.30; ßc_RCWO_ = -0.26). We also found interactions between neuroticism and affective normative commitment (β = 0.24) and between openness and continuance commitment on reduced professional efficacy (β = -0.23), as well as interactions between conscientiousness and continuance commitment on exhaustion.

**Conclusion:**

Although contextual variables were strong prospective predictors and personality traits weak predictors of burnout, the results suggested the importance of the interaction between personality and contextual variables in predicting burnout.

Numerous studies have focused on work stress and burnout in nurses because they work in high-stress environment, which has detrimental effects both on their mental and physical health, productivity and efficacy at work, absenteeism, as well as on patients' outcomes such as increased mortality and patient dissatisfaction (1-3).

Burnout refers to the symptoms of mental/emotional exhaustion caused by chronic job stress (4,5). It manifests itself in the form of exhaustion, depersonalization (cynicism), and the perception of reduced personal efficacy in working with others. Emotional exhaustion refers to feelings of fatigue and loss of energy, depersonalization and detachment from the job, cynicism and mental distancing from service recipients, while reduced professional efficacy refers to feelings of incompetence and a lack of achievement and productivity at work.

The predictors of job burnout are both environmental and individual (5-8). Among frequently examined environmental (organizational) antecedents of burnout are stressors at work such as work overload, role conflict, and role ambiguity. Increased demands at work were strongly related to all components of burnout, and especially to emotional exhaustion (5-8). Rather scarce studies of personality effects found that almost all of 5-factor personality dimensions were related to burnout, although the relations between them were not always strong and consistent (9). However, neuroticism proved to be more strongly and consistently related to burnout than other 5-factor dimensions. Other studies also found positive relations between neuroticism and all three components of burnout (10-15). On the other hand, extraversion was mainly negatively related to burnout (12,14,16), and in some studies negative relations were also found between agreeableness and one or two of burnout dimensions (15,17-19). Conscientiousness was negatively related to emotional exhaustion and reduced professional efficacy and positively to depersonalization, while the relations between openness and burnout dimensions appeared less consistent (20-22).

However, most of the above mentioned studies have cross-sectional designs, meaning that personality dimensions and burnout were examined at the same time, which could result in higher correlations between them. Furthermore, many studies examined burnout in relation to attitudes toward work, most frequently work satisfaction, job involvement, and organizational commitment. Organizational commitment is defined as a degree to which a person identifies himself or herself with the organization and its goals (23). The model of organizational commitment that received considerable empirical support identified 3 components: affective (value-based), normative (obligation-based), and continuance (based on an assessment of costs and benefits) (24). Organizational commitment serves as a protective factor from negative health outcomes and decreases negative effects of stressors on burnout (25).

Although most of the explanatory models of burnout explained it as the outcome of the transaction of environmental and personality variables (26), most often the effects of only one set of variables, organizational (situational) or individual (dispositional), have been examined in a single study. With respect to the evidence that personality influences how people react to stressful situation in their workplace (27), it seems plausible to assume that besides direct effects of personality on one hand, and environmental variables on the other, environmental variables could also moderate the effects of personality on burnout. However, some authors stressed the need for more research on organizational and individual factors that may have direct effects or serve as moderators or buffers of burnout (28,29). Consequently, present study examines the direct effects of both individual and organizational factors, as well as moderating effects of organizational factors on professional burnout in hospital nurses. We examined the direct effects of 5-factor personality variables, and direct and moderating effects of organizational stress and attitudes toward work on 3 components of burnout among hospital nurses measured 4 years later. It was hypothesized that 5-factor personality traits would be predictors of burnout dimensions, and specifically neuroticism was expected to be positive, while extraversion, agreeableness, and conscientiousness negative predictors of burnout. We also tested the possibility that organizational stress would be positive, and affective-normative commitment negative prospective predictor of burnout components. Organizational stress and attitudes toward work (affective-normative commitment and continuance commitment) would be moderators of the effects of personality variables on burnout components.

## Methods

### Participants

A total of 214 registered female hospital nurses selected from 19 (out of 24) wards of the only Clinical Hospital in Rijeka participated in the first part of the study. The study was carried out at two measurement points, in the fall of 2004 (Time 1) and in the fall of 2008 (Time 2). Recruitment to the study was done by ward manager who chose 214 volunteers for the first measurement. The final number of participants was 118, and the reasons why 96 nurses dropped out at Time 2 were the change of work, sick leave, maternity leave, or retirement. The newly employed nurses between Time 1 and Time 2 were excluded from the study mainly because there were very few of them. The age of nurses who participated at both measurement points ranged from 27 to 58 years (mean ± standard deviation 36.47 ± 7.02). The majority had secondary education (68.4%), with their overall working time ranging from 1 to 39 years (mean ± standard deviation 2.49 ± 7.82), and the mean time of working at the same department was 11.64 years (standard deviation 11.63 years).

### Procedure

Questionnaires were administrated individually or in small groups of participants at the beginning of their working day. The study was carried out by previously well prepared psychology students. Participation was voluntary and anonymous (nurses wrote their own codes which they had to remember until Time 2). Participants were given as much time as possible to complete the questionnaires. Hospital review board approved the study protocol. Also the informed consent document was designed to provide information to potential participants.

At the Time 1, Big Five Inventory, Perceived Organizational Stress Inventory, and Organizational Commitment Questionnaire were applied, and Maslach Burnout Inventory at Time 2. Data collection at both measurements lasted a few weeks and only at Time 2 a reminder was sent to the participants.

### Predictor variables

Table 1 presents descriptive characteristics of the instruments used in the study.

**Table 1:** Prospective predictors of professional burnout in hospital nurses and internal reliability coefficients (Cronbach α) of the measures used in the present study

	Mean ± standard deviation	Cronbach α*
Predictor variables:		
extraversion	28.29 ± 4.35	0.64
agreeableness	35.36 ± 4.54	0.67
conscientiousness	37.30 ± 4.02	0.69
neuroticism	20.29 ± 5.19	0.75
openness	35.43 ± 5.03	0.69
role conflict and work overload	27.19 ± 6.07	0.81
affective-normative commitment	34.37 ± 7.21	0.83
continuance commitment	20.67 ± 3.44	0.61
Criterion variables:		
exhaustion	22.69 ± 13.62	0.89
depersonalization	4.88 ± 6.01	0.89
reduced professional efficacy	11.16 ± 8.63	0.78

*Coefficient of internal reliability Cronbach α.

*Personality measure.* Big Five Inventory (BFI) (30) was used to allow quick and efficient assessment of 5-factor personality dimensions. It consists of 44 items – short phrases assessing the most prototypical traits associated with each of the personality dimensions. These are extraversion (eg, “I see myself as someone who is outgoing, sociable”), agreeableness (eg, “I see myself as somebody who is helpful and unselfish with others”), conscientiousness (eg, “I see myself as someone who is reliable worker”), neuroticism (eg, “I see myself as someone who worries a lot”), and openness to experience (eg, “I see myself as someone who is curious about many different things”). Answers were scored on a 5-point scale (1 – strongly disagree; 5 – strongly agree). Despite its brevity, the BFI proved to have good psychometric properties. The coefficients of internal reliability (Cronbach α) of the BFI scales ranged from 0.75 to 0.90 in a Canadian sample (25) and from 0.64 to 0.80 in a Croatian sample (31,32).

*Perceived organizational stress.* Perceived Organizational Stress Inventory was developed by combining items of several well known questionnaires measuring work overload, role conflict, and role ambiguity as predictors of stress at work (33). It consist of 15 items and for each of them participants assessed the frequency of occurrence of a stressful work situation on a five-point scale (1 – never, 5 – almost always). Principle axes factor analysis with Varimax rotation indicates the existence of two factors, the first one being work role conflict and work overload (9 items) (eg, “I do not have enough time to do all my tasks at work”) and the second one role ambiguity (6 items) (eg, “It is completely ambiguous how to do some tasks at my work”). Because role ambiguity showed very low internal reliability coefficient (Cronbach α = 0.49), it was omitted from further analyses and only one variable of organizational stress was retained.

*Organizational commitment.* The organizational commitment questionnaire (24) was translated and adapted to Croatian language (34). It consists of 18 items measuring different aspects of commitment to work organization. Answers are scored on a 5-point scale (1 – definitely disagree; 5 – strongly agree), with higher scores indicating higher commitment. Krapić et al (32) reported the existence of 3 factors: affective (eg, “This hospital means a lot to me”), continuance (eg, “At this moment it would be very hard for me to leave the hospital I work at, even if I wanted to”), and normative commitment (eg, “I owe much to this hospital”), but factor analysis on the participants of the present study indicated the existence of 2 factors; affective-normative and continuance commitment, that explained 31.70% of common variance.

### Criterion variables

*Burnout inventory.* Maslach Burnout Inventory (MBI) (35) consists of 22 items and measures 3 components of burnout: emotional exhaustion (9 items), depersonalization (5 items), and reduced professional efficacy (8 items). Each item is scored on a seven-point scale (0 – never to 6 – every day). High score on this questionnaire is represented by higher score on emotional exhaustion and depersonalization dimension and lower score on reduced professional efficacy dimension. The emotional exhaustion scale measures the degree to which respondents feel overextended (eg, “I feel emotionally drained from my work“) and the depersonalization scale measures the extent to which respondents exhibit an intensive or dehumanized attitude toward service recipients (eg, “I've become more callous toward people since I took this job”). The reduced professional efficacy scale assesses respondents’ feelings of competence and success in their jobs (eg, “I feel I'm positively influencing other people's lives through my work”). In order to follow the results more easily, this scale was reversed. As confirmatory factor analyses of this inventory on Croatian language confirmed its three-factor structure (36), the same structure was also used in the present study.

### Statistical analysis

Data were analyzed by SPSS 15.0 (SPSS Inc., Chicago, IL, USA) for Windows Evaluation Version. First, differences between the nurses who participated at T1 and T2 and those who dropped out at T2 were calculated. Two groups of nurses differed in age (*t* = 3.00, df = 210, *P* = 0.003), agreeableness (*t* = 2.12, df = 212, *P* = 0.035), and continuance commitment (*t* = 2.06, df = 212, *P* = 0.041). Nurses who participated in both parts of the study were older (M_1_ = 33.25, M_1,2_ = 36.44) had lower results on agreeableness (M_1_ = 36.08, M_1,2_ = 34.77) and higher on continuance commitment (M_1_ = 19.63, M_1,2_ = 20.64).

Correlations between variables measured at T1 and correlations of these variables with 3 components of professional burnout measured at T2 were calculated using Pearson correlation coefficients. A set of hierarchical regression analyses was performed to assess the predictive value of 5-factor personality traits, perceived organizational stress, and organizational commitment measured at T1 for 3 components of professional burnout (emotional exhaustion, depersonalization, and reduced professional efficacy) 4 years later, as well as interactions between personality traits and either organizational stress variable (work conflict and role overload) or attitudes toward work (affective-normative commitment and continuance commitment).

In order to examine the predictive value of 5-factor personality dimensions, perceived organizational stress, and organizational commitment on 3 dimensions of professional burnout, 3 groups of hierarchical regression analyses were performed, the main reason for 3 groups of analyses being to avoid too many variables in one analysis. In the first group of regression analyses, 5-factor personality traits were entered as predictors in the first step, perceived role conflict and work overload in the second, and interaction between personality traits and role conflict and work overload in the third. In the second group, 5-factor personality traits were entered as predictors in the first step, affective-normative commitment in the second, and the interaction between 5-factor personality traits and affective-normative commitment in the third. Finally, in the third group, 5-factor personality traits were entered in the first step, continuance commitment in the second, and the interaction between 5-factor personality traits and continuance commitment in the third. Personality traits were always entered in the first step because they represent broad basic biological dispositions and compared with the variables entered in the second step they are not much prone to change. All the regression models were tested for multicollinearity to check that the predictors were not too highly correlated with each other. For all 3 regression models, we checked the variance inflation factor as an index of possible multicollinearity. The highest variance inflation factor values from the present study were lower than 2.5, indicating that the correlation between the predictor variables was not too high.

## Results

The correlations among 5-factor personality traits, perceived organizational stress, and organizational commitment variables measured at Time 1 (Table 2) showed that 5-factor personality dimensions correlated with each other from -0.41 (*P* < 0.010) between neuroticism and agreeableness to 0.40 (*P* < 0.010) between conscientiousness and agreeableness (Table 2). Considering the relations between personality traits and perceived organizational stress, only neuroticism was positively related to role conflict and work overload. Furthermore, neuroticism was negatively and conscientiousness and agreeableness positively related to affective-normative commitment, while none of the 5-factor personality dimensions were related to continuance commitment.

**Table 2:** Correlations between personality traits, organizational stress, and commitment in hospital nurses at first measurement point (Time 1)

	Coefficients of correlations (*P*) for						
Predictors	extraversion	agreeableness	conscientiousness	neuroticism	openness	role conflict and work overload	affective-normative commitment
Agreeableness	-0.11 (0.239)						
Conscientiousness	0.23 (0.011)	0.40 (0.000)					
Neuroticism	-0.25 (0.006)	-0.41 (0.000)	-0.36 (0.000)				
Openness	0.21 (0.022)	0.11 (0.243)	0.28 (0.002)	-0.17 (0.059)			
Role conflict and work overload	-0.11 (0.249)	-0.16 (0.083)	-0.15 (0.103)	0.30 (0.001)	-0.15 (0.104)		
Affective-normative commitment	-0.03 (0.738)	0.28 (0.002)	0.19 (0.041)	-0.24 (0.009)	-0.10 (0.288)	-0.17 (0.060)	
Continuance commitment	0.14 (0.123)	-0.02 (0.871)	0.02 (0.840)	-0.01 (0.900)	-0.01 (0.962)	-0.06 (0.543)	0.18 (0.054)

The correlations between 5-factor personality traits, perceived organizational stress, and organizational commitment with 3 elements of professional burnout variables measured at Time 1 and those measured at Time 2 were computed (Table 3). They show that 5-factor personality traits were not significantly related to burnout, with the exception of significant negative correlations of agreeableness and openness to reduced professional efficacy. Role conflict and work overload was significantly positively related to all dimensions of burnout, affective-normative commitment significantly but negatively to all 3 dimensions, while continuance commitment was not related to any dimension.

**Table 3:** Correlations of personality traits, organizational stress, and commitment with three components of professional burnout in hospital nurses

Variables measured at Time 1	Coefficients of correlations at Time 2 (*P*) for:		
Personality measures:	exhaustion	depersonalization	reduced professional efficacy
extraversion	0.13 (0.150)	0.01 (0.922)	-0.03 (0.763)
agreeableness	-0.15 (0.118)	-0.15 (0.116)	-0.27 (0.003)
conscientiousness	-0.01 (0.941)	-0.05 (0.619)	-0.11 (0.227)
neuroticism	0.13 (0.168)	0.11 (0.236)	0.15 (0.109)
openness	0.03 (0.729)	0.05 (0.622)	-0.19 (0.043)
Perceived organizational stress:			
role conflict and work overload	0.43 (0.000)	0.21 (0.021)	0.20 (0.032)
Organizational commitment:			
affective-normative commitment	-0.23 (0.014)	-0.33 (0.000)	-0.32 (0.000)
continuance commitment	0.00 (0.998)	0.00 (0.994)	0.04 (0.636)

Table 4 presents the results of 3 regression analyses in which 5-factor personality traits were entered as predictors in the first step, perceived organizational stress in the second, and interaction between personality traits and perceived organizational stress in the third.

**Table 4:** The results of hierarchical regression analyses with personality traits and organizational stress in hospital nurses as predictors

Predictor variables (Time 1)	β for criterion variables at Time 2 (*P*):		
	exhaustion	depersonalization	reduced professional efficacy
1. Step – personality traits:			
extraversion	0.17 (0.093)	0.01 (0.948)	-0.04 (0.687)
agreeableness	-0.06 (0.534)	-0.13 (0.242)	-0.28 (0.010)
conscientiousness	0.07 (0.527)	0.05 (0.658)	0.13 (0.233)
neuroticism	0.07 (0.534)	0.06 (0.624)	0.05 (0.661)
openness to experience	0.05 (0.606)	0.07 (0.465)	-0.16 (0.093)
R^2^ (coefficient of multiple determination)*	0.05 (0.307)	0.03 (0.644)	0.10 (0.033)
2. Step – perceived organizational stress:			
role conflict and work overload	0.47 (0.000)	0.23 (0.022)	0.17 (0.069)
R^2^ (coefficient of multiple determination)*	0.23 (0.000)	0.07 (0.260)	0.12 (0.026)
Δ R^2†^	0.18 (0.000)	0.04 (0.039)	0.02 (0.131)
3. Step – personality traits × perceived organizational stress			
extraversion × role conflict and work overload	0.02 (0.804)	0.01 (0.931)	0.01 (0.926)
agreeableness × role conflict and work overload	0.02 (0.886)	0.16 (0.246)	-0.02 (0.887)
conscientiousness × role conflict and work overload	-0.14 (0.234)	-0.23 (0.092)	-0.26 (0.042)
neuroticism × role conflict and work overload	-0.05 (0.649)	-0.03 (0.823)	-0.30 (0.012)
openness to experience × role conflict and work overload	0.07 (0.492)	0.08 (0.450)	-0.07 (0.493)
R^2^ (coefficient of multiple determination)*	0.24 (0.001)	0.09 (0.456)	0.19 (0.018)
Δ R^2†^	0.02 (0.784)	0.03 (0.666)	0.07 (0.121)

*Proportion of variance in the criterion variable explained by predictor variable.

†Difference in R^2^ between the second and first step, and third and second step of the analyses.

**Figure 1:**
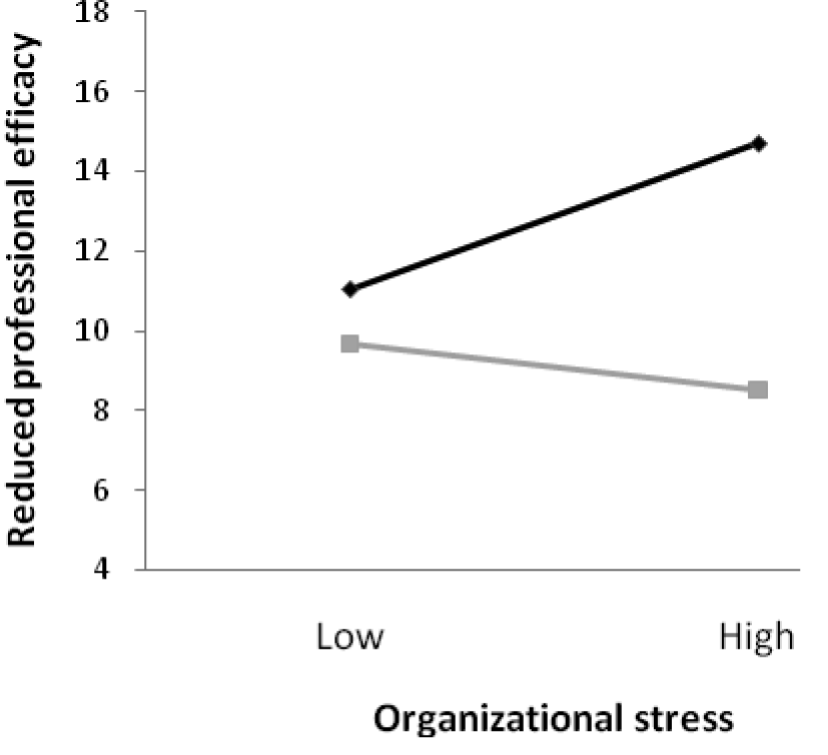
Reduced professional efficacy according to organizational stress intensity in nurses lower and higher on conscientiousness. Black line – nurses lower on conscientiousness; gray line – nurses higher on conscientiousness.

Five-factor personality dimensions as a group significantly predicted only reduced professional efficacy (10%), with agreeableness as a single significant negative predictor of this dimension of burnout. After controlling for 5-factor personality traits in the first step of the analyses, role conflict and work overload additionally significantly and positively predicted emotional exhaustion (ΔR^2^ = 0.18) and depersonalization (ΔR^2^ = 0.04). As a group, variables from the third step did not additionally explain the variance of all 3 dimensions of burnout, but the interactions between conscientiousness and role conflict and work overload, as well as neuroticism and role conflict and work overload, appeared as significant negative predictors of reduced professional efficacy. All 3 groups of predictor variables explained 24% variance of emotional exhaustion and 19% variance of reduced professional efficacy, which was significant, but they did not significantly predict depersonalization (9%). Figure 1 presents the interaction between conscientiousness and role conflict and work overload on reduced professional efficacy.

The perception of reduced professional efficacy increased in nurses lower on conscientiousness in situations of higher organizational stress, while it slightly decreased in nurses higher on conscientiousness in situations of higher organizational stress. 

**Figure 2:**
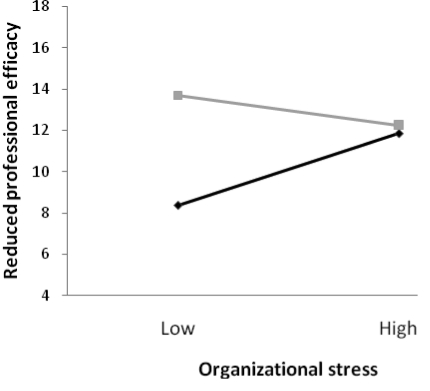
Reduced professional efficacy according to the intensity of role conflict and work overload in nurses lower and higher on neuroticism. Black line – nurses lower on neuroticism; gray line – nurses with higher on neuroticism.

Figure 2 shows the interaction between neuroticism and role conflict and work overload on reduced professional efficacy. The perception of reduced professional efficacy increased in nurses lower on neuroticism (higher stability) in situations of higher organizational stress, while it decreased in nurses higher in neuroticism in situations of higher organizational stress.

Table 5 presents the results of regression analyses in which 5-factor personality traits were entered as predictors in the first step, affective-normative commitment in the second, and interaction between 5-factor personality traits and affective-normative commitment in the third step.

**Table 5:** The results of hierarchical regression analyses with personality traits and affective-normative commitment in hospital nurses as predictors

Predictor variables (Time 1)	β for criterion variables at Time 2 (*P*):		
	exhaustion	depersonalization	reduced professional efficacy
1. Step – personality traits:			
extraversion	0.09 (0.420)	-0.03 (0.784)	-0.11 (0.294)
agreeableness	-0.09 (0.473)	-0.12 (0.324)	-0.26 (0.026)
conscientiousness	0.10 (0.400)	0.07 (0.562)	0.10 (0.325)
neuroticism	0.09 (0.422)	0.03 (0.814)	-0.02 (0.874)
openness to experience	0.03 (0.777)	0.02 (0.850)	-0.18 (0.060)
R^2^ (coefficient of multiple determination)*	0.05 (0.307)	0.03 (0.644)	0.10 (0.033)
2. Step – organizational commitment:			
affective-normative commitment	-0.21 (0.046)	-0.29 (0.008)	-0.30 (0.003)
R^2^ (coefficient of multiple determination) *	0.08 (0.132)	0.11 (0.034)	0.18 (0.010)
Δ R^2†^	0.03 (0.052)	0.08 (0.002)	0.08 (0.001)
3. Step – personality traits × organizational commitment:			
extraversion × affective-normative commitment	0.14 (0.169)	0.01 (0.945)	-0.01 (0.956)
agreeableness × affective-normative commitment	0.09 (0.490)	-0.06 (0.646)	0.05 (0.706)
conscientiousness × affective-normative commitment	-0.11 (0.393)	0.06 (0.640)	-0.05 (0.703)
neuroticism × affective-normative commitment	0.20 (0.099)	0.04 (0.721)	0.24 (0.033)
openness to experience × affective-normative commitment	0.06 (0.571)	-0.07 (0.605)	0.12 (0.322)
R^2^ (coefficient of multiple determination) *	0.14 (0.141)	0.12 (0.223)	0.23 (0.002)
Δ R^2†^	0.05 (0.284)	0.01 (0.976)	0.05 (0.284)

*Proportion of variance in the criterion variable explained by predictor variable.

†Difference in R^2^ between the second and first step of the analyses and third and second step of the analyses.

After controlling for 5-factor personality dimensions, affective-normative commitment was additionally significantly related to the variance of depersonalization (ΔR^2^ = 0.08) and reduced professional efficacy (ΔR^2^ = 0.08), and for these 2 criterion variables it appeared as a negative predictor. Variables from the third step as a group did not significantly explain any of the three criteria, although the interaction between neuroticism and affective-normative commitment appeared as a single significant positive prospective predictor. All three groups of predictor variables explained 23% variance of reduced professional efficacy, which was significant, but they did not significantly predict either emotional exhaustion (14%) or depersonalization (12%).

**Figure 3:**
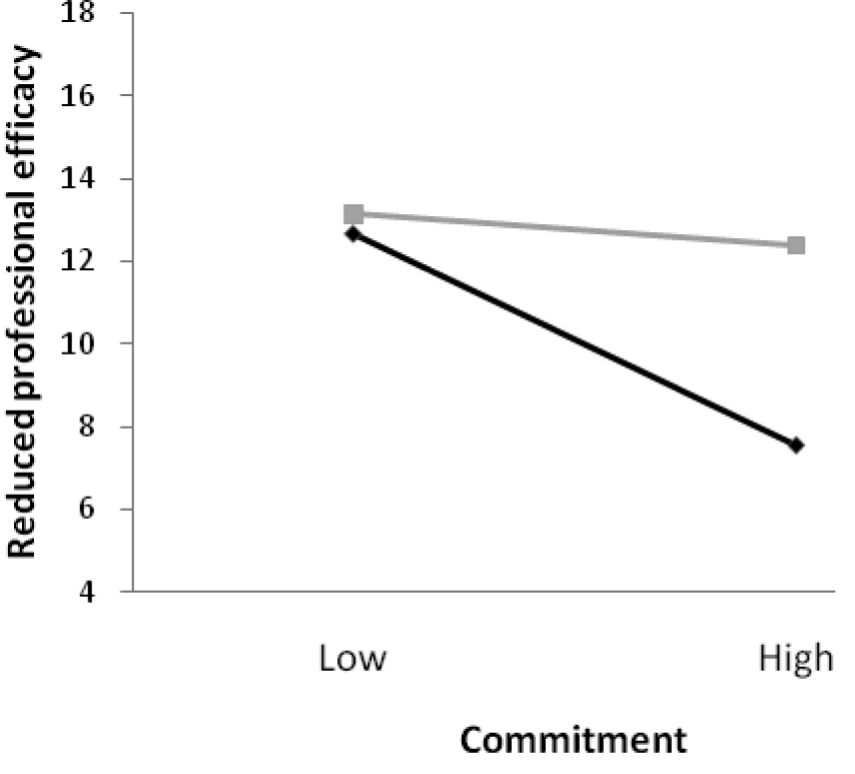
Reduced professional efficacy according to the intensity of affective-normative commitment in nurses lower and higher on neuroticism. Black line – nurses lower on neuroticism; gray line – nurses higher on neuroticism.

Figure 3 shows the interaction between neuroticism and affective-normative commitment on reduced professional efficacy.

The perception of reduced professional efficacy decreased in nurses higher on affective-normative commitment and especially in those with low neuroticism (more stable), while in those higher on neuroticism it remained almost the same notwithstanding the degree of their affective-normative commitment.

Table 6 presents the results of the third group of hierarchical regression analyses in which 5-factor personality traits were entered as predictors in the first step, continuance commitment in the second, and interaction between 5-factor personality traits and continuance commitment in the third step.

**Table 6:** The results of hierarchical regression analyses with personality traits and continuance commitment as predictors

Predictor variables (Time 1)	β for criterion variables at Time 2 (*P*):		
	exhaustion	depersonalization	reduced professional efficacy
1. Step – personality traits:			
extraversion	0.14 (0.209)	-0.05 (0.654)	-0.11 (0.283)
agreeableness	-0.07 (0.552)	-0.09 (0.425)	-0.23 (0.041)
conscientiousness	0.09 (0.423)	0.05 (0.658)	0.09 (0.387)
neuroticism	0.16 (0.141)	0.07 (0.537)	-0.01 (0.934)
openness to experience	-0.05 (0.655)	0.01 (0.924)	-0.21 (0.037)
R^2^ (coefficient of multiple determination)*	0.05 (0.307)	0.03 (0.644)	0.10 (0.033)
2. Step – organizational commitment:			
continuance commitment	-0.10 (0.312)	-0.06 (0.580)	0.02 (0.849)
R^2^ (coefficient of multiple determination)*	0.05 (0.422)	0.03 (0.764)	0.10 (0.056)
Δ R^2†^	0.00 (0.820)	0.00 (0.991)	0.02 (0.631)
3. Step – personality traits × organizational commitment:			
extraversion × continuance commitment	-0.17 (0.101)	-0.20 (0.061)	-0.13 (0.194)
agreeableness × continuance commitment	-0.11 (0.333)	-0.21 (0.100)	-0.15 (0.199)
conscientiousness × continuance commitment	0.30 (0.014)	0.21 (0.094)	0.02 (0.862)
neuroticism × continuance commitment	0.04 (0.704)	-0.09 (0.412)	-0.18 (0.090)
openness to experience × continuance commitment	0.01 (0.888)	-0.07 (0.504)	-0.23 (0.025)
R^2^ (coefficient of multiple determination)*	0.12 (0.206)	0.10 (0.422)	0.18 (0.028)
Δ R^2†^	0.07 (0.135)	0.07 (0.169)	0.07 (0.099)

*Proportion of variance in the criterion variable explained by predictor variable.

†Difference in R^2^ between the second and first step of the analyses and third and second step of the analyses.

Although 5-factor personality traits as a group significantly prospectively predicted only reduced professional efficacy (R^2^ = 0.10), with agreeableness and openness as significant negative prospective predictors of reduced professional efficacy, they did not significantly predict other 2 dimensions of burnout. After controlling for 5-factor personality traits, in the second step of analyses, continuance commitment alone did not predict any of the 3 dimensions of burnout directly, although in the third step two significant interactions appeared, one between conscientiousness and continuance commitment on exhaustion and the other between openness and continuance commitment on reduced professional efficacy.

All three groups of predictor variables did not significantly explain the variance of any of the 3 burnout dimensions (emotional exhaustion, 12%; depersonalization, 10%; reduced professional efficacy, 18%).

The first interaction showed that in nurses with lower conscientiousness scores emotional exhaustion significantly decreased when they were higher on commitment (mean ± standard deviation 23.57 ± 15.54) compared to those lower on commitment (mean ± standard deviation 20.62 ± 13.26), while in nurses with higher conscientiousness scores the feeling of exhaustion increased when they were higher on continuance commitment (mean ± standard deviation 21.73 ± 13.13) in comparison with lower continuance commitment (mean ± standard deviation 24.72 ± 12.70). Second, the interaction between openness to experience and continuance commitment showed that nurses lower on openness to experience increased their perception of reduced professional efficacy when their continuance commitment was higher (mean ± standard deviation 14.00 ± 9.44) in comparison to lower commitment (mean ± standard deviation 11.42 ± 8.52), while those higher on openness to experience decreased their perception of reduced professional efficacy when higher on continuance commitment (mean ± standard deviation 7.74.45 ± 7.43) in comparison with lower continuance commitment (mean ± standard deviation 11.45 ± 8.14).

## Discussion

We showed that out of the 5-factor personality traits, only agreeableness and openness were negatively related to only one dimension of burnout – reduced professional efficacy. On the other hand, organizational stress measured as role conflict and work overload showed significant positive correlations with all 3 dimensions, and affective-normative commitment negative relations with all 3 dimensions. However, continuance commitment did not have any relations to burnout at all (Table 3).

Although hierarchical regression analyses showed that 5-factor personality traits as a group prospectively predicted only one dimension of burnout – reduced professional efficacy, they also indicated that out of the 5-factor personality traits agreeableness, and to a lesser extent openness, lowered the perception of reduced professional efficacy in hospital nurses. These results are in accord with the results found for teachers (17) and emphasize the importance of agreeableness as a protective factor from reducing personal efficacy at least in human service occupations such as nursing and teaching. Because work success in nurses depends on their tendency to help and care for their patents, it could be expected that agreeableness would be related to performance (37).

Regarding stress in organizations operationalized here as role conflict and work overload, the results showed that it predicted two elements of burnout: emotional exhaustion and depersonalization (Table 4). It could be said that the concept of role conflict and work overload encompasses 2 of the 6 areas of work life considered as central correlates of burnout, workload, and control (5). Namely, workload may result from too many demands at work and lack of skills or inclination for a certain type of work requirements. All of these exhaust the energy, and therefore the findings showing that workload was related to emotional exhaustion are not surprising (5,38). On the other hand, role conflict may raise uncertainty because of the lack of perceived control over the resources or lack of sufficient authority for performing the job successfully (6). Therefore, a person may distance himself or herself from service recipients by behaving as though they are impersonal objects. It is also likely that health care services in our country have reduced resources and increased demands for hospital nurses. Thus, persistent stressors at work may make nurses question their own abilities and worth, as well as make them feel less confirmed and valued, which may lead to depersonalization.

Our results showed that affective-normative commitment was a prospective protective factor of all 3 components of burnout. The importance of organizational commitment has been discussed widely and it has been considered as protective factor from negative effects of burnout (39). By definition, affectively committed individuals remain with their organization because they wish to do so and normatively committed remain because they feel they ought to (40,41). Therefore, affectively normatively committed employees are highly motivated to do their work effectively either because of their feelings toward their job or organization, which gives them energy to persist in stressful circumstances and protects them from burnout. Given that organizational commitment is relatively stable and not a subject to daily fluctuations, and because of its protective role in burnout, its relationship to burnout should be examined in future studies in more detail.

The obtained interactions between personality traits and organizational stress showed that organizational stress moderated the relations between conscientiousness, neuroticism, and the perception of reduced professional efficacy. These results further explain the mechanisms through which conscientiousness and neuroticism affect work performance as these two personality variables have been previously considered to be critical traits for success in the work place and predictors of performance motivation (42,43). Namely, the results showed that nurses lower on conscientiousness were at risk of increasing the perception of reduced personal efficacy in situations of higher role conflict and work overload, while in those higher on this trait the perception of reduced professional efficacy even slightly decreased (Figure 1). Having in mind the characteristics of conscientiousness (competence, order, dutifulness, achievement striving, self-discipline, and deliberation), it seems plausible that nurses higher on conscientiousness would not be so sensitive to stressful work conditions when self-evaluation of their professional accomplishment is concerned, while those lower on this trait would be more prone to feel as their efficacy at work was diminished.

In nurses higher on neuroticism, the perception of reduced professional efficacy remained almost the same regardless of the intensities of organizational stress, while in those lower on neuroticism the perception of reduced professional efficacy increased in situations of high role conflict and work overload. In situations of higher organizational stress nurses with higher and lower neuroticism scores perceived the same level of reduced personal efficacy. Although research has generally confirmed that individuals high on neuroticism usually judge their occupational self-efficacy as lower than individuals low on this dimension and that individuals low on neuroticism generally feel in control of their environment (22), this study might suggest that more stable nurses were more responsive to the stressful situations at work and that the perception of their own professional competence and efficacy was more prone to change according to working conditions. Because of the small sample size in this study, more studies are needed to confirm and clarify these results. Furthermore, the interaction of neuroticism and affective-normative commitment on reduced professional efficacy indicated that the perception of reduced professional efficacy decreased in nurses higher on affective-normative commitment, and especially in those with lower neuroticism (more stable), while in those higher in neuroticism it remained almost the same notwithstanding the intensity of their affective-normative commitment (Figure 3). An emotionally stable individual who at the same time has strong feelings of emotional attachment to his or her job and a desire to act in ways that are consistent with membership of the occupation could be expected to have a reliable perception of personal accomplishment, and therefore these results might have been expected.

Additionally, nurses higher on conscientiousness showed increased exhaustion when higher on continuance commitment, while those lower on this dimension showed decreased exhaustion when higher on continuance commitment. These results seem to be plausible because, by definition, continuance commitment refers to the perception of high costs associated with leaving organization and therefore it may be expected that low dutifulness, orderliness, and self-discipline found in low-conscientious people would make them more resistant to emotional exhaustion in situations in which they perceive a need to stay in the organization whether they like it or not. On the other hand, in the same situations, individuals high on conscientiousness, dutiful, and self-disciplined, would feel more exhausted. Also, nurses higher on openness to experience had lower perception of reduced professional efficacy when higher on continuance commitment, while those lower on openness to experience showed increased perception of reduced professional efficacy when their continuance commitment was higher. We can assume that individuals high on openness to experience, described as ready to re-examine their own values, appreciative of new ideas, and rich in imagination, would re-appraise their reasons for staying, cope better, and be more resourceful in situation of perceived need to stay in their organization because of high costs of leaving it. This could result in more positive evaluation of their professional efficacy. On the other hand, people low on openness and high on continuance commitment would perceive their work as something they have to do and therefore be more prone to evaluate their working efficacy negatively.

Generally, the results suggest that although 5-factor personality traits as a group were weak and organizational stress and affective-normative commitment were much stronger predictors of burnout, burnout should be considered as a result of the transactions between contextual and personality variables.

The limitations of this study are exclusive use of self-report as well as small sample size and sample attrition due to drop-out at Time 2. Also, the study could have benefited more with its design if burnout was measured at Time 1 and controlled for in regression analyses. Additionally, significant differences between the nurses who were included at both measurement points and those who dropped out were found. The two groups differed in age, agreeableness, and continuance commitment, with the nurses who participated in both parts of the study being older, less agreeable, and higher on continuance commitment. These differences could have influenced the results.

On the other hand, the inclusion of both personality and environmental variables, as well as the interactions between them in a single study should be considered as its relative advantages.

Our results strongly indicate that those hospital departments in which nurses perceive high organizational stress might be at a higher risk for professional burnout. There is a need for organizational interventions aimed at prevention and reduction of organizational stress. Also, affective-normative commitment in nurses should be seen as protective factor of burnout, and some interventions could be done to increase this type of commitment. Furthermore, within the selection procedure for hospital nurses, out of 5-factor personality traits, agreeableness and to a lesser extent openness, should be taken into account as prospective protective factors against development of burnout symptoms. And finally, the obtained interactions of neuroticism, conscientiousness, and openness with organizational stress, affective-normative, and continuance commitment should be considered as combinations of variables worth paying attention to when planning organizational interventions for nurses most prone to burnout.
